# Maintenance of non-consciously presented information engages the prefrontal cortex

**DOI:** 10.3389/fnhum.2014.00938

**Published:** 2014-11-21

**Authors:** Fredrik Bergström, Johan Eriksson

**Affiliations:** ^1^Umeå center for Functional Brain Imaging (UFBI)Umeå, Sweden; ^2^Department of Integrative Medical Biology, Physiology Section, Umeå UniversityUmeå, Sweden

**Keywords:** non-conscious, durability, attention, conscious experience, perception, working memory

## Abstract

Conscious processing is generally seen as required for flexible and willful actions, as well as for tasks that require durable information maintenance. Here we present research that questions the assumption that only consciously perceived information is durable (>500 ms). Using the attentional blink (AB) phenomenon, we rendered otherwise relatively clearly perceived letters non-conscious. In a first experiment we systematically manipulated the delay between stimulus presentation and response, for the purpose of estimating the durability of non-conscious perceptual representations. For items reported not seen, we found that behavioral performance was better than chance across intervals up to 15 s. In a second experiment we used fMRI to investigate the neural correlates underlying the maintenance of non-conscious perceptual representations. Critically, the relatively long delay period demonstrated in experiment 1 enabled isolation of the signal change specifically related to the maintenance period, separate from stimulus presentation and response. We found sustained BOLD signal change in the right mid-lateral prefrontal cortex, orbitofrontal cortex, and crus II of the cerebellum during maintenance of non-consciously perceived information. These findings are consistent with the controversial claim that working-memory mechanisms are involved in the short-term maintenance of non-conscious perceptual representations.

## Introduction

The functional complexity of the human brain enables us to perceive and interact with our environment in a flexible and deliberate manner. However, despite our intuition to the contrary, we only consciously experience a fraction of the accompanying processes. We thus have the capacity to perceive more information than we can consciously experience, with the consequence that some perceived information about the external environment remains non-conscious, as demonstrated by phenomena like masking (Dehaene et al., 2001) and the attentional blink (AB; Luck et al., 1996).

Higher-level cognitive functions, associated with frontal and parietal cortical regions, have traditionally been considered the exclusive product of conscious processes, while non-conscious processes have been considered limited to automatic, lower-level functions (Koch and Crick, 2001). Correspondingly, neuroimaging studies investigating the neural correlates of conscious experiences have often found that activity in the prefrontal and parietal network (PPN) correlates with conscious perception (Rees et al., 2002; Naghavi and Nyberg, 2005). Based on the frequent involvement of the PPN in conscious perception, the Global Neuronal Workspace (GNW) model states that widespread and recurrent prefrontal and parietal activity determines if information is consciously experienced or not. According to the model, long-distance axons of the PPN, together with thalamocortical loops, form a “global workspace” that interconnects many specialized, automatic, and (otherwise) non-conscious processors. Non-conscious information is hypothesized to become conscious once it is globally broadcast via the PPN and thereby available to many brain regions, enabling depth of processing, more flexible use of information, and durable (>500 ms) representations in working memory and long-term memory (Dehaene and Changeux, 2011; Dehaene et al., 2014).The effects of non-conscious perception have been investigated and hotly debated during the past century (see Kouider and Dehaene, 2007, for review). Recent discoveries suggest that several functions previously associated uniquely with conscious processing can take place after non-conscious perception, and in some cases, activate parts of the PPN, e.g., cognitive control (Lau and Passingham, 2007), flexibility and context-specificity (Wokke et al., 2011), monetary motivation (Pessiglione et al., 2007), and error detection (Logan and Crump, 2010). Most recently, durable (up to 5 s) non-conscious perceptual representations have been demonstrated (Soto et al., 2011), challenging the common notion that non-conscious representations are extremely short-lived.

To investigate the durability of non-conscious representations we here used the AB paradigm as a way to manipulate the conscious experience of seeing a particular stimulus. As the AB effect is not consistent across trials (for a given set of parameters, participants will see a target stimulus on some trials and not see it on others), the AB phenomenon is a useful tool for creating conditions with identical experimental parameters, but with differing conscious experiences. Furthermore, the AB is known to enable relatively long non-conscious presentation durations, e.g., 100 ms (Martens and Wyble, 2010), compared with up to 50 ms for masking (Greenwald et al., 1996). The AB paradigm therefore has the potential to elicit relatively strong non-conscious brain activity (Sergent et al., 2005), and possibly more durable representations.

In a first behavioral experiment we manipulated the delay durations between unseen stimuli presentations and responses to estimate the longevity of non-conscious representations, establishing that non-conscious representations can last for up to 15 s. In a second experiment we used fMRI with a similar paradigm to investigate the neural correlates underlying the maintenance of non-conscious representations. Critically, the relatively long interval between stimulus and response enabled a within-trial separation of BOLD signal related to different trial components (stimulus presentation, delay period, and response), similar to the approach used in neuroimaging research on working memory (Curtis and D’Esposito, 2003).

According to the GNW model there should be no working-memory involvement during processing of non-conscious representations. Contrary to this prediction however, recent research has suggested that working memory operations could account for the durable retention of non-conscious representations (Soto et al., 2011; Pan et al., 2013; Soto and Silvanto, 2014). Furthermore, Dutta et al. (2014) have demonstrated BOLD signal increase in PPN during a delayed cue-target orientation discrimination task with non-conscious sample presentations. However, given the sluggishness of the BOLD signal and that the delay period used by Dutta et al. was short (1.5 s), it is unclear if the signal change was related to maintenance or to stimulus and/or response processing. If working-memory mechanisms indeed are responsible for the maintenance of non-conscious representations there should be sustained BOLD signal change in brain regions characteristically involved in working memory during the delay period, specifically, frontal and parietal cortex related to executive processes and temporal integration of previously attained perceptual knowledge and its prospective use (Cabeza and Nyberg, 2000; Wager and Smith, 2003; Fuster, 2009; Sreenivasan et al., 2014).

## Materials and methods

### Participants

For experiment 1 (behavioral experiment) 24 participants were recruited from the Umeå University campus area. All participants had normal or corrected-to-normal vision, gave written informed consent, and were paid for participation. Participants were excluded if they failed to comply with instructions (two participants for systematically pressing the same response instead of guessing), or if they had significantly different reported perceptual awareness ratings of target stimulus as a function of time (one participant). Twenty one participants (18–39 year age range, *M* = 24 years, 13 female) were thus included in the statistical analyses.

For experiment 2 (fMRI experiment) 27 participants were recruited from the Umeå University campus area. All participants were right handed and had normal or corrected-to-normal vision, gave written informed consent, and were paid for participation. The experiment was approved by the ethics committee at the University Hospital of Northern Sweden. Participants were excluded if they failed to comply with instructions (one participant for systematically pressing the same response instead of guessing), or if they had significantly different reported perceptual awareness ratings as a function of time (no participant excluded). Twenty six participants (21–29 year age range, *M* = 24 years, 15 female) were thus included in the statistical analyses.

### Stimuli and procedure

**Figure 1:**
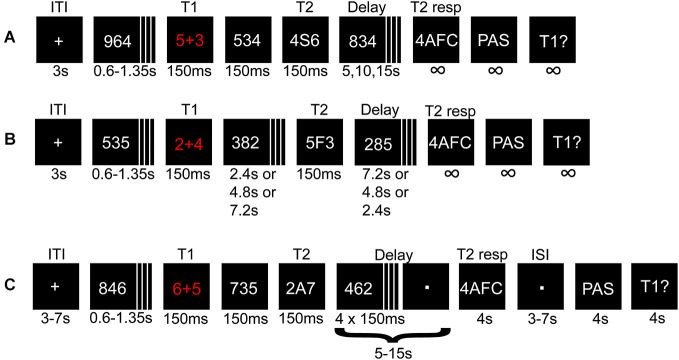
**Illustration depicting the procedure for experiment 1 and 2**. A math task (T1) and a letter (T2) flanked by distracters were presented in a rapid serial visual presentation (RSVP). The solution to T1, and T2 identity, were held in memory during a variable delay until probed for responses. **(A)** Short- and **(B)** long-lag trials in experiment 1 and the pre-fMRI session. In **(B)** the variable delays before and after T2 presentation were adjusted such that T2 appeared early, in the middle, or at the end of the RSVP. **(C)** Illustration of a short-lag trial in the fMRI session.

In experiment 1, two targets were presented in a rapid serial visual presentation (RSVP) sequence consisting of three-digit distractors (Figure 1A). The first target (T1) was an addition task displayed in red, which the participants were instructed to solve immediately and to retain the answer until prompted to respond. The second target (T2) was a letter (A, S, D, or F) flanked by two randomly assigned digits. By presenting T1 and T2 in a specific time sequence, visibility of T2 is severely reduced. This phenomenon is usually explained as an effect of attentional processing of T1 that hinders processing of T2, either by depleting resources or through attentional control mechanisms (Raymond et al., 1992; Martens and Wyble, 2010). A key goal of the present experimental paradigm was to allow for a relatively long T2 stimulus duration (e.g., 133 ms) by generating a strong AB effect. To this end T1 consisted of an attentionally demanding, but mathematically simple addition task, under the assumption that a more demanding T1 enhances the AB effect (Martens and Wyble, 2010). Stimulus duration was initially set to 133 ms and then adjusted online (shifted up or down between blocks in steps of one display refresh rate (60 Hz); each of six blocks consisted of 42–60 trials, depending on T1 performance (incorrect T1 response automatically added a trial until a correct T1 response or the upper trial limit was reached), totaling in 252–360 trials) to ensure an approximate 50/50 distribution between seen/unseen trials despite individual differences.

A critical parameter for the AB is the lag between T1 and T2, such that if the lag is too short or too long, the effect is attenuated or canceled. We were here mainly interested in parameters that cause the AB to occur in approximately 50% of the trials to allow a comparison of conscious vs. non-conscious processing. However, to verify the phenomenon as such with the current experimental protocol, we used both short (strong AB effect) and long (weak AB effect) lags between T1 and T2. Each short-lag RSVP started with a central crosshair for 3000 ms after which 4–9 distractors were presented, followed by T1, one intervening distractor, and T2. Distractors were then displayed until 5, 10, or 15 s had passed from T2 presentation. The long-lag RSVPs (*n* = 36) were randomly interspersed with short-lag trials (*n* = 180), differing in the number of distractors between T1 and T2, and in delay time (always 10 s; Figure 1B). During trials with the longest T1-T2 lag, T2 was presented at the end of the RSVP. Thus, the participants had to attend the entire RSVP, during which they did not know whether they missed a short-lag T2 or if a long-lag T2 was to be presented at the very end.

After each RSVP participants answered three queries: (i) a four-alternative forced-choice (4AFC) task regarding T2 identity; (ii) to what degree they had a subjective experience of seeing T2; (iii) the answer to T1. The conscious experience of seeing T2 or not was judged on a four-point perceptual awareness scale (PAS; Overgaard et al., 2006; Sandberg et al., 2010). The PAS scale descriptions used were: (1) no visual experience of T2; (2) vague visual experience of T2; (3) almost clear visual experience of T2; and (4) clear visual experience of T2. All ratings above 1 were treated as indicating conscious perception. When T2 was unseen the participants had been instructed to guess when prompted regarding T2 identity by responding with the first letter that came to mind. To create a reference condition for the subjective experience of not seeing T2, there were also 36 trials without a T2 (replaced by a distractor). Therefore, trials with and without T2 that were given PAS ratings of 1 shared the same (lack of) subjective experience of T2.

Experiment 2 consisted of a pre-fMRI session (four blocks totaling 124–144 trials depending on T1 performance and/or if the upper trial limit was reached; 84 short-lag, 20 long-lag, and 20 T2-absent trials) and an fMRI session (two runs, each run consisted of two blocks totaling 116 trials; 80 short-lag and 36 T2-absent trials), and used a similar procedure as for experiment 1 with the following changes. The pre-fMRI session was modified by using three instead of four steps in the PAS scale, because combining “clear or almost clear experience” (steps 3 and 4) was easier and more intuitive to use for the participants, without losing the important distinction between “no experience” and “vague experience”. The pre-fMRI session enabled us to screen for unsuitable participants, e.g., “non-blinkers” (Martens and Wyble, 2010), and to adjust the individual stimulus durations for approximate 50/50 distribution of seen/unseen trials before the fMRI session, although stimulus durations were also adjusted during the fMRI session. The fMRI session had some additional changes (Figure 1C): (i) added jitter to the inter-trial interval (3–7 s), delay-period (5–15 s in steps of 1 s instead of 5 s), and the inter-stimulus interval (3–7 s) between the 4AFC and PAS response, to reduce correlations between components of the statistical model; (ii) the delay period consisted of passively viewing a dot, but four distractors remained after T2 presentation to uphold the AB effect and overwrite any iconic memory representations; and (iii) response-time limits of 4 s for all responses.

### fMRI acquisition

The fMRI session in experiment 2 was conducted at 3T with a GE 3 Tesla Discovery MR750 scanner with a 32-channel receive-only head coil. Each subject underwent one session with two functional runs (784 volumes each) of scanning using a T2^*^-weighted gradient echo pulse sequence, echo planar imaging, field of view = 25 cm, matrix size = 96 × 96, slice thickness = 2.9 mm, 37 slices with no inter-slice skip and an ASSET acceleration factor of 2. The volumes covered the whole cerebrum and most of the cerebellum, the acquisition orientation was oblique axial and aligned with the anterior and posterior commissure, and was scanned in interleaved order with TE = 30 ms, TR = 2 s, flip angle = 90°. Between the two functional runs a high-resolution T1-weighted structural image was collected FSPGR with TE = 3.2 ms, TR = 8.2 ms, TI = 450 ms, and flip angle = 12°.

### Data processing and statistical analysis

For the behavioral results of both experiments, only trials where T1 was answered correctly were used in the analyses, because if T1 was not processed there would likely not be an AB during T2. For response times a two SD cut-off was used for each condition (PAS > 1 and PAS = 1) and participant separately.

The software used for processing and analysis of fMRI data was SPM8 (Welcome Trust Centre for Neuroimaging, London, UK), run in Matlab 7.11 (Mathworks, Inc., Sherborn, MA, USA). Before preprocessing a manual quality control was conducted using in-house software. Preprocessing was done in the following order: slice-timing correction to the first slice using Fourier phase-shift interpolation method, head-motion correction with unwarping of B0 distortions, DARTEL normalization (Ashburner, 2007) using a 12-parameter affine transformation model to MNI anatomical space, and an 8 mm FWHM Gaussian smoothing. DARTEL normalization and smoothing was applied on the contrast images after intrasubject model estimation.

For intrasubject modeling a General Linear Model (GLM) with restricted maximum likelihood estimation was used. The model consisted of the following regressors of interest: trial epochs (stimulus presentation, delay, and response)-by-trial type (short-lag or T2 absent)-by-T1 accuracy (correct or incorrect)-by-PAS rating (1, 2, or 3), and inter-trial interval. Missed 4AFC responses (because of time limit), head motion (six parameters) and physiological noise (six parameters) estimated with tCompCor (temporal variation in white matter and cerebral spinal fluid; Behzadi et al., 2007), were included as nuisance regressors. All regressors except for head motion and physiological noise were convolved with the “canonical” hemodynamic response function. The high-pass filter had a cut-off at 128 s, and the autocorrelation model was global AR (1).

For each individual and each trial epoch (stimulus presentation, delay, and response), the following conditions were compared: T2-seen > T2-absent and T2-unseen > T2-absent. Average signal change across conditions during each of the three trial epochs relative to a low-level baseline (ITI) was defined as (T2-seen + T2-unseen + T2-absent)/3 > ITI. Model estimations from each individual were taken into second-level random-effects analyses (one-sample *t*-tests) to account for inter-individual variability. The statistical inferences were made on the whole brain with *p* ≤ 0.001 uncorrected for multiple comparisons, cluster extent ≥20.

## Results

### Behavioral results

The average T1 performance was 82% for experiment 1, 86% for the pre-fMRI session, and 80% for the fMRI session. The average proportion of unseen T2s (PAS = 1 given correct T1) when T2 was present were 45% for experiment 1, 37% for the pre-fMRI session, and 32% for the fMRI session. The average proportion of false alarms were 12% for experiment 1, 12% for the pre-fMRI session, and 25% for the fMRI session.

There was a significant difference in T2 performance between short- and long-lag trials, (experiment 1: *F*_(1, 20)_ = 74.70, *p* < 0.001; experiment 2: *F*_(1, 25)_ = 30.94, *p* < 0.001), thereby replicating previous research on the AB in that the T1-T2 time interval had a high impact on T2 performance.

In experiment 1, there was a main effect of T2 visibility (T2-seen and T2-unseen trials) on T2 performance, but no main effect of delay time (Table 1). There was a significant visibility-by-delay time interaction, such that seen T2 performance declined over time, whereas unseen T2 performance did not. Critically, performance on unseen T2 was significantly better than chance (0.25) at all three time points (Table 1). A second analysis only on T2-unseen (PAS = 1) trials revealed no significant main effect of delay time (Table 1). Response times for T2-seen trials were significantly shorter than T2-unseen trials (*t*_(1, 20)_ = −3.18, *p* = 0.005) and T2-absent trials (*t*_(1, 20)_ = −2.57, *p* = 0.02). Response times for T2-unseen trials were not significantly different from T2-absent trials (*t*_(1, 20)_ = −0.39, *p* = 0.70).

**Table 1:** **Behavioral results**.

	F/t (df)	*p*-value	PAS = 1		PAS > 1	
			M (SE)	95% Cl		M (SE)	95% Cl	
				LL	UL		LL	UL
Experiment 1
T2 visibility	129.98 (1, 20)	**< 0.001**	**0.34 (0.02)**	0.30	0.38	**0.75 (0.04)**	0.68	0.83
Delay	1.02 (2, 40)	0.37						
T2 visibility*delay	4.15 (2, 40)	**0.02**						
T2 acc. 5 s			**0.32 (0.02)**	0.28	0.37	**0.79 (0.03)**	0.73	0.86
T2 acc. 10 s			**0.36 (0.03)**	0.30	0.41	**0.73 (0.05)**	0.62	0.84
T2 acc. 15 s			**0.33 (0.03)**	0.28	0.39	**0.74 (0.03)**	0.66	0.81
T2 delay (PAS = 1)	1.14 (2, 40)	0.33						
T2 response times	−3.17 (1, 20)	**0.005**	1962 (88)	1780	2145	1706 (89)	1521	1890
Experiment 2: Pre-fMRI
T2 visibility	148.25 (1, 25)	**< 0.001**	**0.33 (0.02)**	0.28	0.39	**0.78 (0.03)**	0.70	0.85
Delay	2.09 (2, 50)	0.13						
T2 visibility*delay	0.14 (2, 50)	0.87						
T2 acc. 5 s			**0.34 (0.03)**	0.28	0.41	**0.80 (0.03)**	0.74	0.87
T2 acc. 10 s			**0.34 (0.04)**	0.27	0.42	**0.78 (0.04)**	0.70	0.86
T2 acc. 15 s			0.31 (0.04)	0.23	0.39	**0.74 (0.04)**	0.66	0.83
T2 delay (PAS = 1)	0.41 (2, 50)	0.66						
T2 response times	−3.43 (1, 25)	**0.002**	1619 (143)	1326	1913	1228 (57)	1112	1345
Experiment 2: fMRI
T2 visibility	49.73 (1, 25)	**< 0.001**	**0.41 (0.42)**	0.32	0.50	**0.75 (0.05)**	0.65	0.85
Delay	1.55 (1, 25)	0.23						
T2 visibility*delay	0.72 (2, 50)	0.49						
T2 acc. 5–8 s			**0.35 (0.05)**	0.26	0.46	**0.74 (0.05)**	0.63	0.86
T2 acc. 9–11 s			**0.47 (0.06)**	0.34	0.60	**0.76 (0.06)**	0.64	0.88
T2 acc. 12–15 s			**0.39 (0.06)**	0.27	0.51	**0.74 (0.05)**	0.64	0.84
T2 delay (PAS = 1)	1.56 (2, 50)	0.23						
T2 response times	−2.64 (1, 25)	**0.01**	1193 (70)	1049	1337	1066 (43)	977	1155

Note: Significant values are in boldface. Cl = confidence interval; LL = lower limit; UL = upper limit; T2 visibility = the main effect of visibility/PAS on T2 performance; Delay = the main effect of delay time on T2 performance; T2 visibility*delay = the interaction effect between visibility and delay time on T2 performance; T2 acc. = T2 performance after X s delay time; T2 delay (PAS = 1) = main effect of delay time on unseen T2 performance only; T2 response times = t-test comparison between seen and unseen T2 response times (ms). T-values are reported for response time comparison, and F-values for all other comparisons.

All behavioral results in experiment 1 were replicated in experiment 2 for the pre-fMRI and fMRI session with two exceptions: (i) there was no significant T2 visibility-by-delay time interaction in experiment 2; and (ii) T2-unseen performance was at chance-level during the third time point in the pre-fMRI session (Table 1).

In experiment 2, response times for T2-seen trials were significantly shorter than T2-unseen trials (pre-fMRI: *t*_(1, 25)_ = −3.43, *p* = 0.002; fMRI: *t*_(1, 25)_ = −2.64, *p* = 0.01) and T2-absent trials (pre-fMRI: *t*_(1, 25)_ = −3.76, *p* = 0.001; fMRI: *t*_(1, 25)_ = −3.46, *p* = 0.002). Response times for T2-unseen trials were not significantly different from T2-absent trials (pre-fMRI: *t*_(1, 25)_ = −1.28, *p* = 0.21; fMRI: *t*_(1, 25)_ = −1.56, *p* = 0.13).

### fMRI results

#### Using the attentional blink to investigate memory

The AB phenomenon has been used extensively in previous research to investigate attention and also conscious experience. It is less commonly used to investigate aspects of memory. Here, we have used the AB to manipulate conscious perception but have designed the experiment similar to protocols investigating working memory, with a stimulus presentation, followed by a short delay, followed by a probe. Similar to previous neuroimaging research on working memory, we used multiple regression to identify BOLD signal change specifically related to different within-trial components (stimulus presentation, delay, and probe). To verify this approach, we first compared each trial epoch with a low-level baseline (the inter-trial interval), averaged across the three conditions (the participants were required to keep information online during the delay period even for T2-absent trials, as T1 was present in all trials).

**Figure 2:**
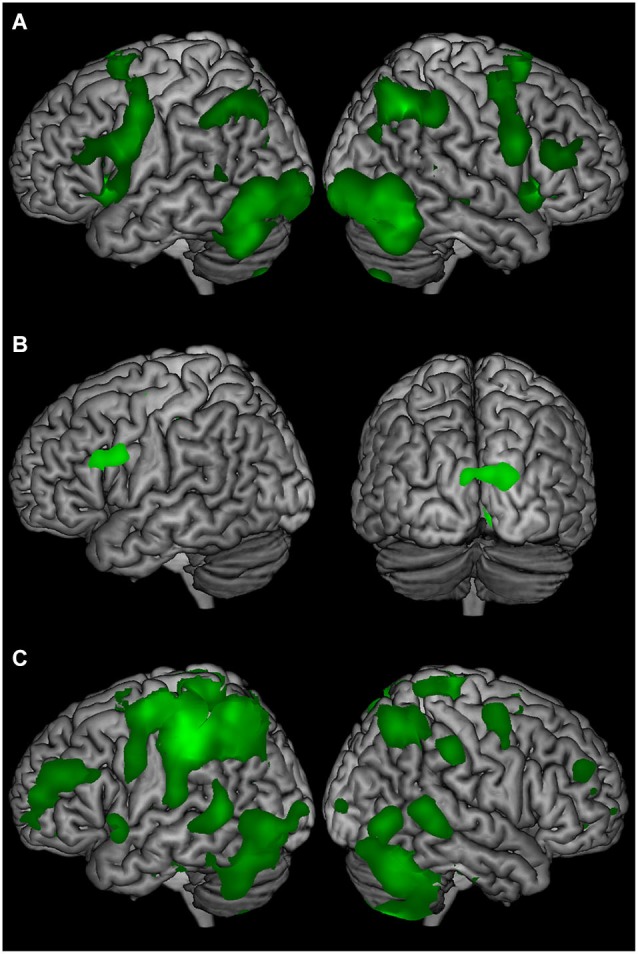
**The average BOLD signal change of T2-seen, T2-unseen, and T2-absent trials compared to a low-level baseline (ITI) for (A) stimulus presentation, (B) the delay period, and (C) the response epochs**.

Comparing stimulus presentation with the low-level baseline revealed BOLD signal change in widespread frontal, parieto-temporal, and cerebellum regions bilaterally (Figure 2A). The delay-period comparison against the low-level baseline revealed sustained BOLD signal change in the left inferior frontal gyrus, and bilateral occipital cortex (Figure 2B). Comparing the response and the low-level baseline revealed bilateral BOLD signal change in the frontal, parietal, temporal, and occipital cortex, and cerebellum (Figure 2C).

#### Comparing T2-present and T2-absent trials

**Figure 3:**
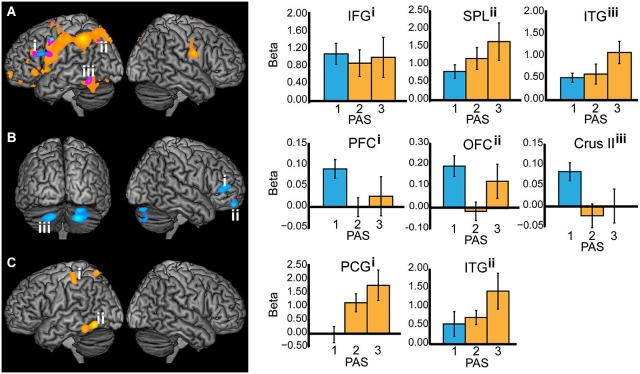
**All working memory epochs with T2-seen > T2-absent trials in orange, T2-unseen > T2-absent trials in blue, and the overlap in purple**. The Y-axis: Beta values, X-axis: PAS = perceptual awareness scale, bar colors correspond to the PAS classification of T2-seen and T2-unseen trials, error bars: standard error of the mean. **(A)** Stimulus presentation (IFG = inferior frontal gyrus, *X* = −46, *Y* = 28, *Z* = 24; SPL = superior parietal lobule, *X* = −26, *Y* = −68, *Z* = 44; ITG = inferior temporal gyrus, *X* = −52, *Y* = −54, *Z* = −18). **(B)** Delay period—displayed at *p* < 0.005 for illustrative purposes (PFC = prefrontal cortex, *X* = 50, *Y* = 42, *Z* = 8; OFC = orbitofrontal cortex, *X* = 20, *Y* = 58, *Z* = −22; Crus II in the cerebellum, *X* = −20, *Y* = −74, *Z* = −38. **(C)** Response (PCG = postcentral gyrus, *X* = −42, *Y* = −30, *Z* = 52; ITG = inferior temporal gyrus, *X* = −58, *Y* = −62, *Z* = −12).

Comparing T2-seen with T2-absent trials during T2 stimulus-presentation revealed wide-spread BOLD signal change, most notably in the left inferior temporal gyrus, parieto-occipital, and frontal cortex (Figure 3A), but also in the right parietal cortex and the left hippocampus. There was a similarly wide-spread, but much less pronounced, pattern of BOLD signal change limited to the left inferior temporal gyrus, superior parietal lobule, inferior frontal gyrus, and precentral gyrus when comparing T2-unseen with T2-absent trials.

When comparing the delay period of T2-seen with T2-absent trials no significant BOLD signal change was found. The comparison between the delay period of T2-unseen and T2-absent trials revealed sustained BOLD signal change in the right mid-lateral prefrontal cortex (mid-lateral PFC; crossing inferior and middle frontal gyrus BA 45/46), right orbitofrontal cortex (OFC), and bilateral cerebellum (crus II). Comparing the delay period of T2-unseen with T2 seen trials revealed a cluster in the mid-lateral PFC (*t* = −3.76) that overlapped with the cluster found when comparing T2-unseen with T2-absent. To investigate the relationship between BOLD signal change during the delay period and task performance we correlated beta values from the right mid-lateral PFC and OFC with unseen T2 performance across participants. There was no significant relation between regional BOLD signal change in mid-lateral PFC and task performance (*r*_(25)_ = −0.12, *p* = 0.58) or OFC and task performance (*r*_(25)_ = −0.07, *p* = 0.75).

When comparing the responses for T2-seen with T2-absent trials BOLD signal change was found in the left inferior temporal gyrus, postcentral gyrus, and superior parietal lobule (Figure 3C). Comparing T2-unseen and T2-absent trial responses revealed no significant BOLD signal change.

## Discussion

In contrast to common belief, we have shown that non-consciously presented perceptual information can be durable, here lasting with unaffected strength for at least 15 s. This result replicate and extend previous findings of durable perceptual representations by Hesselmann et al. (2011) of 4 s, and Soto et al. (2011) of 5 s, and further challenge durability as a hallmark for conscious experience. The sustained BOLD signal change in mid-lateral PFC during maintenance of the non-consciously perceived information is consistent with working memory. Our findings are in line with Dutta et al. (2014) who found that BOLD signal change in DLPFC over entire trials of masked information predicted memory performance. Critically, however, our within-trial separation of BOLD signal confirmed that sustained BOLD signal change in the PFC occurs during maintenance of non-conscious information.

Several different memory mechanisms, such as working memory, priming, or iconic memory, could in principle be responsible for durable non-conscious representations. Although the cortical aspects of iconic memory (fragile visual short-term memory) can retain information for at least 4 s, it is easily overwritten by task-irrelevant distracters (Sligte et al., 2008). Similar to Soto et al. (2011), the current AB paradigm used irrelevant distracters after stimulus-presentation. This, combined with the current delay of up to 15 s, makes iconic memory an unlikely explanation.

Furthermore, the sustained BOLD signal change found in right mid-lateral PFC, OFC, and cerebellum during the maintenance of non-consciously perceived information is inconsistent with priming. The exact mechanisms of priming are still unclear and may depend on task, material, and whether the material is masked or not (Henson, 2003). However, priming effects are likely either residual neural activity and/or latent neural changes (e.g., long-term potentiation) that facilitates or inhibits subsequent processing (Grill-Spector et al., 2006; Marsolek et al., 2010). Neither residual neural activity nor latent neural changes would elicit a sustained BOLD signal change over the entire delay period.

Thus, only working memory remains as a possible explanation for the durable non-conscious perceptual representations. Although working memory is commonly seen as intimately linked with conscious experience (Baars and Franklin, 2003; Dehaene and Changeux, 2011; Baddeley, 2012), it has been suggested on theoretical grounds that working memory indeed can operate non-consciously, as below-threshold activity (Fuster, 1995). Other recent empirical studies also support the notion of non-conscious working memory. Soto et al. (2011) have shown that 1–2 non-consciously presented items can be maintained during a distractor-filled delay of up to 5 s. Dutta et al. (2014) linked performance on a delayed task with non-consciously presented information to BOLD signal change in the PFC. Furthermore, they showed that transcranial direct current stimulation of the PFC modulated performance, demonstrating that the PFC is causally involved in such delayed performance. Pan et al. (2013) demonstrated that when a non-consciously maintained item matched an interocularly suppressed item, the latter had prior entry into conscious awareness compared to non-matching items. Critically, none of the results above can be explained by priming mechanisms, because Soto et al. (2011) and Dutta et al. (2014) used delayed cue-target orientation discrimination tasks where cue and target never matched, and Pan et al. (2013) did several control experiments to show that mere exposure to the masked stimulus was not enough for prior entry, it had to be actively maintained.

Neuroimaging studies of humans and single-unit recordings in primates have revealed that working memory maintenance is associated with sustained neural activity in lateral PFC and posterior regions. Prefrontal cortex activity has been interpreted as representing a preparatory action set, while posterior activity represents the memory content (Cabeza and Nyberg, 2000; Curtis and D’Esposito, 2003; Fuster, 2009; Sreenivasan et al., 2014). Consistent with our findings and non-conscious working memory, recent findings suggest that non-conscious information can activate task sets (Reuss et al., 2011) and lead to lateral prefrontal BOLD signal change (Lau and Passingham, 2007).

However, it is unclear why the sustained BOLD signal change in the right PFC did not correlate with memory performance, as might have been expected (Pessoa et al., 2002; Sakai et al., 2002; Wager et al., 2014). Different lateral PFC regions have been related to different executive functions during working memory (Wager and Smith, 2003; Nee et al., 2013), and it is conceivable that not all functions necessarily predicts performance. Progressively rostral regions of the PFC seem to support more abstract representations and more complex rules (Fuster, 2008; Badre and D’Esposito, 2009). The mid-lateral PFC signal change could therefore be related to task set maintenance (Sakai and Passingham, 2003) or preparation for future action (Pochon et al., 2001), while the OFC signal change might relate to maintenance of more abstract representations (Nee and Brown, 2012) or executive control functions related to coordination and simultaneous use of several cognitive processes such as maintenance, manipulation, and monitoring (Owen et al., 2005; Barbey et al., 2011).

The lateral prefrontal cortex has contralateral input and output projections that form closed loops with crus I and II of the cerebellum (Kelly and Strick, 2003; Bostan et al., 2013). Although the exact function of the cerebellum in cognition is unclear, BOLD signal change in crus I and II together with prefrontal cortex have been associated with verbal working memory and executive functions (Stoodley and Schmahmann, 2009).

The comparison of average signal change across conditions with a low-level baseline revealed differences in the left inferior frontal gyrus, and bilaterally in the occipital cortex during maintenance. The signal change in inferior frontal gyrus is consistent with neuroimaging findings related to sub-vocalization during working memory maintenance (Paulesu et al., 1993), which suggest that sub-vocalization was used to remember the consciously perceived T1 and T2. It is unclear why there was no significant BOLD signal change during the delay period when comparing T2-seen and T2-absent trials. Possibly, the difference between consciously maintaining 1 vs. 2 items (T1 vs. T1 + T2) was not big enough to elicit detectable BOLD signal change. The fact that there was a difference during delay between T2-unseen and T2-absent trials therefore suggest that temporary maintenance of information can engage different processes depending on if the information to be maintained is conscious or not. Although consciously perceived verbal information is likely maintained by way of sub-vocalization, it seems unlikely for non-consciously perceived verbal information to be so. Instead, the non-consciously perceived verbal information might be maintained as visual representations. Consciously sub-vocalizing T1 while non-consciously maintaining T2 could therefore act as a distraction or dual-task/process that leads to increased PFC involvement (D’Esposito et al., 1995; Feredoes et al., 2011). Different maintenance processes for conscious and non-conscious representations would explain the stronger BOLD signal change in the right mid-lateral PFC during maintenance of non-consciously compared to consciously perceived information.

For T2-seen compared to T2-absent trials, BOLD signal change was evident in the left inferior temporal gyrus during stimulus presentation and response. This likely reflects representational-level processing of T2, as previous neuroimaging research has demonstrated signal change in inferior temporal gyrus during single letter perception (Flowers et al., 2004; Park et al., 2012). However, there was no significant BOLD signal change in inferior temporal gyrus during the delay period following consciously and non-consciously perceived information. Unit recordings in primates have established that stimulus-specific neurons in sensory regions are temporarily activated during working memory retention (Fuster and Jervey, 1981; Miyashita and Chang, 1988; Miller and Desimone, 1994), but human neuroimaging findings have not been as consistent. Several working-memory studies with letter-tasks did not reveal inferior temporal gyrus involvement during maintenance and manipulation of letters (Cabeza and Nyberg, 2000). Although the signal change in stimulus-specific sensory regions fail to reach the same elevated level as frontal regions during maintenance, it has recently been shown that multivariate pattern analysis can detect stimulus-specific information (Riggall and Postle, 2012). It could therefore be the case that simply maintaining one item in working memory is generally not enough to elicit elevated levels of BOLD signal in stimulus-specific sensory regions.

Consistent with the GNW model and previous findings a comparison between T2-seen and T2-absent trials revealed large and wide-spread BOLD signal change in the left inferior temporal gyrus (Flowers et al., 2004; Park et al., 2012), and PPN (Rees et al., 2002; Naghavi and Nyberg, 2005; Dehaene and Changeux, 2011) during the stimulus presentation. Corresponding BOLD signal change was also found in inferior temporal gyrus during the comparison between T2-unseen and T2-absent trials, which is consistent with previous studies on non-conscious perception (Rees et al., 2002; Marois et al., 2004; Heinzel et al., 2008; Dehaene and Changeux, 2011). However, the wide-spread PPN involvement in T2-unseen compared to T2-absent trials during stimulus presentation is inconsistent with the GNW model. Although previous masking studies have found that non-conscious processing tends to be limited (but not exclusive) to posterior sensory regions (Dehaene et al., 1994, 2001; Kouider et al., 2007), more recent studies have implicated the PPN (Kranczioch et al., 2005; Diaz and McCarthy, 2007) and prefrontal cortex (Lau and Passingham, 2007; Wokke et al., 2011) in non-conscious processing. It is conceivable that our wide-spread BOLD signal change was the result of relatively long stimulus presentation durations (*M* = 129 ms, during fMRI) compared to the 17–50 ms commonly used in masking paradigms. Furthermore, that non-consciously presented information not only can activate, but also maintain durable representations in higher-order regions, such as the mid-lateral PFC and the OFC, for 15 s is inconsistent with the GNW model’s predictions that extended PFC activity and durable maintenance is unique to conscious processing (Dehaene and Changeux, 2011).

An alternative model (to GNW) of conscious experience predicts that neural processes are accompanied by conscious experience if (and only if) the neural activity reaches a certain undefined threshold. Such amplitude models (Fuster, 1995; Zeki, 2001) predict that conscious experience should correlate with higher amplitudes, and has no *a priori* reason to assume that higher-order regions, wide-spread cortical interactions, or higher-level cognitive functions such as working memory should be uniquely reserved for conscious experiences. Instead, amplitude models predict that non-conscious functions, and their underlying neural activity would be similar but weaker, which is what neuroimaging findings seem to indicate (Rees et al., 2002; Lau and Passingham, 2006; Diaz and McCarthy, 2007; Wokke et al., 2011).

Comparing T2-seen with T2-absent trials during the response revealed BOLD signal change in the left inferior temporal gyrus, postcentral gyrus, and superior parietal lobule. The inferior temporal gyrus signal change is consistent with previous research, where signal change usually involves a transient peak in both prefrontal and posterior regions during stimulus presentation and response epochs, and a lower, sustained BOLD signal change during the maintenance epoch (Druzgal and D’Esposito, 2003). The contrast between T2-unseen and T2-absent trials did not reveal a significant BOLD signal difference. Interestingly, the nominal BOLD signal change in inferior temporal gyrus during response was comparable to the (significant) signal change during stimulus presentation (Figure 3). Thus, it seems that the inferior temporal gyrus signal change was non-significant due to higher variability rather than amplitude, which is in line with recent proposals of (low) variability as a hallmark of conscious processes (Schurger et al., 2010).

There are several valid approaches to measure and operationally define conscious experience and the lack thereof. We have here used a subjective measure of awareness. Compared with objective measures, subjective measures are more liberal and risk overestimating the extent of non-conscious processing. However, the more conservative objective measures may instead underestimate the effect of non-conscious processing by (miss) attributing them as conscious (Merikle et al., 2001). Possibly, using conservative measures of conscious experience may have biased previous findings that show non-conscious processes to be short-lived. The particular measure used here (“The Perceptual Awareness Scale”) has been shown superior to other subjective measures such as confidence ratings (Cheesman and Merikle, 1986) and post-decision wagering (Persaud et al., 2007) in terms of sensitivity and exhaustiveness (Sandberg et al., 2010).

The use of a four-alternative forced-choice task with fixed response options opens up the possibility that the participants decided how to respond during the delay instead of when prompted. This is, however, not consistent with the response-time data. If participants already decided what letter to guess before prompted, then the response times for unseen T2s should be the same as for seen T2s, because in both cases the participants would know their prospective response in advance. Instead, if the participants decided what to guess when prompted, the unseen trials should have a slower response time than seen trials to account for extra deliberation time, which was the case. Indeed, there was no significant difference in response time between T2-unseen and T2-absent trials. Furthermore, the sustained BOLD signal change found when comparing the T2-unseen and T2-absent trials cannot be explained by such behavior. Given that the lack of perceptual awareness of T2 was identical for both conditions, the (deliberate) strategy must have been identical as well.

In sum, we have demonstrated that non-conscious perceptual representations can last for up to 15 s despite irrelevant distracters, and argue that this effect is best explained in terms of (non-conscious) working-memory mechanisms. Most notably, we found sustained BOLD signal change in the right mid-lateral PFC and OFC during the delay period. In addition, we found widespread frontal and parieto-temporal BOLD signal change during non-conscious perception. Although it is too early to say whether these durable non-conscious representations can truly be understood as working memory processes and not some other form of non-conscious memory, the current findings combined with recent similar research are compelling (Soto and Silvanto, 2014). Important next steps will be to convincingly show that a stimulus-specific representation is actively maintained during the delay-period (e.g., by using multivariate pattern classification algorithms), to compare potential functional differences between conscious and non-conscious working-memory operations (e.g., in terms of capacity and/or fidelity), and to determine how conscious and non-conscious working memory operations interact.

## Conflict of interest statement

The authors declare that the research was conducted in the absence of any commercial or financial relationships that could be construed as a potential conflict of interest.
